# Supplementary material for: Predation and fragmentation portrayed in the statistical structure of prey time series
Source: BMC Ecol. 2009 May 6;9:10. doi: 10.1186/1472-6785-9-10 (PMC2689204; doi:10.1186/1472-6785-9-10)
Supplement: Additional file 2 — Voles and related classes ODDox Documentation. ODDox documentation of the agent-based model (ALMaSS) applied by Hendrichsen et al. The documentation is started by activating main.html. [file 1472-6785-9-10-S2.zip › Vole_ODDox/struct_farm_event.html]

ALMaSS ODDox: FarmEvent Struct Reference

- Main Page
- Related Pages
- Classes
- Files

- Alphabetical List
- Class List
- Class Hierarchy
- Class Members

# FarmEvent Struct Reference

`#include <farm.h>`

List of all members.

---

## Detailed Description

A struct to hold the information required to trigger a farm event.

|  |  |
| --- | --- |
|  |  |
| Public Member Functions | |
|  | FarmEvent (TTypesOfVegetation a\_event, LE \*a\_field, int a\_todo, long a\_run, bool a\_lock, int a\_start, bool a\_first\_year, TTypesOfVegetation a\_nextcrop) |
| Public Attributes | |
| TTypesOfVegetation | m\_event |
| LE \* | m\_field |
| bool | m\_first\_year |
| bool | m\_lock |
| TTypesOfVegetation | m\_next\_tov |
| long | m\_run |
| int | m\_startday |
| int | m\_todo |

---

## Constructor & Destructor Documentation

|  |  |  |  |  |
| --- | --- | --- | --- | --- |
| FarmEvent::FarmEvent | ( | TTypesOfVegetation | *a\_event*, |  |
|  |  | LE \* | *a\_field*, |  |
|  |  | int | *a\_todo*, |  |
|  |  | long | *a\_run*, |  |
|  |  | bool | *a\_lock*, |  |
|  |  | int | *a\_start*, |  |
|  |  | bool | *a\_first\_year*, |  |
|  |  | TTypesOfVegetation | *a\_nextcrop* |  |
|  | ) |  |  | `[inline]` |

References m\_event, m\_field, m\_first\_year, m\_lock, m\_next\_tov, m\_run, m\_startday, and m\_todo.

```
00178     {
00179       m_event = a_event; m_field    = a_field;
00180       m_todo  = a_todo;  m_run      = a_run;
00181       m_lock  = a_lock;  m_startday = a_start;
00182       m_first_year = a_first_year;
00183       m_next_tov=a_nextcrop;
00184     }
```

---

## Member Data Documentation

TTypesOfVegetation FarmEvent::m\_event

Referenced by FarmEvent(), and Farm::LeSwitch().

LE\* FarmEvent::m\_field

Referenced by Farm::CheckRotationManagementLoop(), FarmEvent(), Farm::HandleEvents(), and Farm::LeSwitch().

bool FarmEvent::m\_first\_year

Referenced by WinterWheat::Do(), SetAside::Do(), and FarmEvent().

bool FarmEvent::m\_lock

Referenced by WinterWheat::Do(), SetAside::Do(), and FarmEvent().

TTypesOfVegetation FarmEvent::m\_next\_tov

Referenced by FarmEvent().

long FarmEvent::m\_run

Referenced by FarmEvent(), and Farm::LeSwitch().

int FarmEvent::m\_startday

Referenced by WinterWheat::Do(), SetAside::Do(), and FarmEvent().

int FarmEvent::m\_todo

Referenced by WinterWheat::Do(), SetAside::Do(), FarmEvent(), and Farm::LeSwitch().

---

The documentation for this struct was generated from the following file:

- farm.h

---

Generated on Thu Jan 22 14:13:46 2009 for ALMaSS ODDox by 
 1.5.6
